# Anæsthesia in Dental Surgery

**Published:** 1905-06

**Authors:** 


### Bibliography.

ANÆSTHESIA IN DENTAL SURGERY. By Thomas D. Luke, M.B., F.R.C.S.E., Anæsthetist to the Dental Hospital, the Deaconess Hospital, and Instructor in Anæsthetics to the University Surgical Classes, Royal Infirmary, Edinburgh. With twenty-eight Illustrations. Rebman Company, New York, 1903.

This book, while somewhat too limited in the general treatment of a very important branch of practice, is nevertheless one of the most valuable books in its practical application thus far given the dental profession upon the subject of anæsthesia. The book is dedicated to Dr. John Girdwood, of Edinburgh, to whom the dental profession is indebted for many valuable suggestions and improvements.

While this book has been prepared in Scotland, it has been published in New York, probably a wise move on the part of the author, though seemingly too far away from the home of its production.

The author says of it, "The aim of this little work—which, as far as the author knows, is the first of its nature in the field—has been to pass the various anæsthetics and combinations of them used in operative dentistry, as it were, in review before the reader's eye, with a brief description of the properties of some of them, their method of application, advantages, and disadvantages."

The author's extended experience as an anæsthetist naturally leads the reader to expect the book to be a practical manual upon the subject, and in this the reviewer feels he will not be disappointed. It covers all the at present known anæsthetics and analgesics, and, while the author has aimed at brevity, he has given the readers a very satisfactory and instructive book.

The history of anæsthesia, as presented, does full justice to Dr. Horace Wells and also to Dr. William T. Morton, the original discoverers of anæsthesia by inhalation. He also recognizes the services of Dr. James Young Simpson in introducing chloroform, but it is thought more notice of the discoverers of chloroform should have been given than is to be found, very briefly stated, on the first page.

Much space is properly given to nitrous oxide. The author does not regard the after-effects of this anæsthetic of serious import, for he says, "The after-effects of nitrous oxide are usually exceedingly slight and transient. . . . If at all marked, some impurity of the gas may be suspected or the administration may have been faultily conducted." This is probably true, as a rule, but it must not be forgotten that quite serious after-effects are by no means uncommon, frequently assuming serious physical disturbance of a chronic character. This may have been due to the causes stated, but the assertion requires further proof before acceptance.

"Gas and oxygen" claims attention, but the author does not write enthusiastically in regard to this combination, for he says, "We thus find that, while just double the time is required than when using pure $N_2O$, the anæsthesia obtained is only half as long again in duration."

The results attained by the combination of gas with ether do not warrant its general use. The narcosis may be prolonged, but this is not required in the ordinary operation of extraction, while responsibility is greatly increased. The author prefers ethyl chloride and ether to the former. "There is less cyanosis, and anæsthesia is more pleasantly and rapidly produced."

Ethyl bromide is regarded "as well worthy of the dental surgeon's attention, but the author does not devote much space to its consideration. He states that with this anæsthetic "the mortality has been estimated at under 1 in 5000."

Ethyl chloride is given extended consideration; in fact, the author felt obliged to revise his text, as the manuscript was practically completed before he had had an opportunity to test its value. The comparison between the anæsthetic periods of nitrous oxide and ethyl chloride are in favor of the latter. "Dr. McCardie finds, on the average: Time of induction, fifty-one seconds; length of anæsthesia, seventy seconds, but in some cases extending to two or two and a half minutes.

"This compares very favorably with nitrous oxide, which, according to Hewitt, requires 55.9 seconds to induce full anæsthesia."

Space will not permit a further following of the author through "Mouth-gags," "Anæsthetics in Special Cases," "Local Anæsthesia," "Management of Prolonged Extraction Cases," all of which are valuable, but are too briefly considered.

The great difficulty with all experts seems to be to fail to drop sufficiently into detail. They seem to lose sight of the necessity of talking to "the lowest man in the class."

The author's long dissertation on "The Place of Chloroform in Dental Surgery" will be far more valuable to English practitioners than to Americans, for chloroform is very rarely used here in dental operations.

The author gives a list of deaths from nitrous oxide, made up to 1901. These amount to seventeen, with thirteen additional in doubt as to the cause. This, if correct, is certainly a remarkable showing in view of the many thousands constantly inhaling nitrous oxide for minor surgical operations.

"Accidents of Anæsthesia" concludes the book.

While the author might with great advantage extend the text in future editions by entering more into detail, it is thought the book, as written, furnishes the best small book yet published upon the subject. The dental profession has had several works published on nitrous oxide in this country, and able articles upon anæsthesia in various dental publications, but this is the only work covering the entire subject prepared by a practical anæsthetist, and it is therefore of peculiar value and will meet the needs of many in general practice.

